# Bone regeneration by human dental pulp stem cells using a helioxanthin derivative and cell-sheet technology

**DOI:** 10.1186/s13287-018-0783-7

**Published:** 2018-02-01

**Authors:** Yasuyuki Fujii, Yoko Kawase-Koga, Hironori Hojo, Fumiko Yano, Marika Sato, Ung-il Chung, Shinsuke Ohba, Daichi Chikazu

**Affiliations:** 1 Department of Oral and Maxillofacial Surgery, Tokyo Medical University Hospital, 6-7-1 Nishishinjuku, Shinjuku-ku, Tokyo, 160-0023 Japan; 2 Division of Clinical Biotechnology, Graduate School of Medicine, The University of Tokyo, 7-3-1 Hongo, Bunkyo-ku, Tokyo, 113-8655 Japan; 3 Sensory and Motor System Medicine, Graduate School of Medicine, The University of Tokyo, 7-3-1 Hongo, Bunkyo-ku, Tokyo, 113-8655 Japan; 4 Department of Bioengineering, The University of Tokyo Graduate School of Engineering, 7-3-1 Hongo, Bunkyo-ku, Tokyo, 113-8655 Japan

**Keywords:** Dental pulp stem cells, Bone regeneration, Cell-sheet technology, Small compound, Osteogenic differentiation

## Abstract

**Background:**

Human dental pulp stem cells (DPSCs), which have the ability to differentiate into multiple lineages, were recently identified. DPSCs can be collected readily from extracted teeth and are now considered to be a type of mesenchymal stem cell with higher clonogenic and proliferative potential than bone marrow stem cells (BMSCs). Meanwhile, the treatment of severe bone defects, such as fractures, cancers, and congenital abnormalities, remains a great challenge, and novel bone regenerative techniques are highly anticipated. Several studies have previously shown that 4-(4-methoxyphenyl)pyrido[40,30:4,5]thieno[2,3-b]pyridine-2-carboxamide (TH), a helioxanthin derivative, induces osteogenic differentiation of preosteoblastic and mesenchymal cells. However, the osteogenic differentiation activities of TH have only been confirmed in some mouse cell lines. Therefore, in this study, toward the clinical use of TH in humans, we analyzed the effect of TH on the osteogenic differentiation of DPSCs, and the in-vivo osteogenesis ability of TH-induced DPSCs, taking advantage of the simple transplantation system using cell-sheet technology.

**Methods:**

DPSCs were obtained from dental pulp of the wisdom teeth of five healthy patients (18–22 years old) and cultured in regular medium and osteogenic medium with or without TH. To evaluate osteogenesis of TH-induced DPSCs in vivo, we transplanted DPSC sheets into mouse calvaria defects.

**Results:**

We demonstrated that osteogenic conditions with TH induce the osteogenic differentiation of DPSCs more efficiently than those without TH and those with bone morphogenetic protein-2. However, regular medium with TH did not induce the osteogenic differentiation of DPSCs. TH induced osteogenesis in both DPSCs and BMSCs, although the gene expression pattern in DPSCs differed from that in BMSCs up to 14 days after induction with TH. Furthermore, we succeeded in bone regeneration in vivo using DPSC sheets with TH treatment, without using any scaffolds or growth factors.

**Conclusions:**

Our results demonstrate that TH-induced DPSCs are a useful cell source for bone regenerative medicine, and the transplantation of DPSC sheets treated with TH is a convenient scaffold-free method of bone healing.

**Electronic supplementary material:**

The online version of this article (10.1186/s13287-018-0783-7) contains supplementary material, which is available to authorized users.

## Background

Current cell-based therapies are in need of alternative treatment techniques that enable cells to be readily harvested. Recently, dental pulp stem cells (DPSCs) were identified, which were found to differentiate into multiple cell lineages, such as adipogenic, neurogenic, and osteogenic cells [1–3]. DPSCs are now considered to be a type of mesenchymal stem cell (MSC) and demonstrate higher clonogenic and proliferative potential than bone marrow stem cells (BMSCs). Compared with other MSCs, such as BMSCs, DPSCs are very easily isolated from extracted teeth by low-invasive surgery without any ethical issues. Therefore, DPSCs are a promising cell source for regenerative medicine and tissue engineering.

Current treatment methods are unable to fully reconstruct bone defects that are caused by fractures, cancers, congenital abnormalities, and so forth. At present, autogenous or allogeneic bones and artificial materials are used for bone augmentation. However, there are various problems with these grafts, such as the possibility of infection or absorption as well as ethical issues. To overcome such problems, a more simple and efficient method for transplantation is urgently required. Recently, cell-sheet engineering has emerged as a cell-transplantation system that requires no scaffolds or carriers, and has been used for regenerative treatment of the cornea, heart, liver, and so forth [4–6]. Cell sheets can be easily isolated by reducing the temperature of viable cells cultured on the temperature-responsive culture dish surface, with no requirement of digestive enzymes or denaturing treatments [7]. Moreover, for cell-based therapies in regenerative medicine, optimization of the culture conditions regarding proliferation and differentiation is required. Recombinant proteins, such as bone morphogenic protein (BMP), are used clinically to induce new bone formation, and BMPs were reported to have the capacity of osteogenic induction in primary human BMSCs [8]. However, recombinant proteins are expensive and unstable. Furthermore, BMPs play important roles not only in bone formation but also in the development of cancer [9]. In contrast, several studies have shown that small chemical compounds induce bone formation. For example, statins [10], TAK-778 [11], and isoflavone derivatives [12] were reported to stimulate osteogenic differentiation, and we reported previously the helioxanthin derivative 4-(4-methoxyphenyl)pyrido[40,30:4,5]thieno[2,3-b]pyridine-2-carboxamide (TH) to be a small molecule that induces osteogenic differentiation of preosteoblastic cells and mesenchymal cells in vitro [13, 14]. However, the osteogenic differentiation activities of TH were confirmed only in specific cell lines, such as C3H10T1/2 cells and MC3T3-E1 cells, and these effects on human primary cells remain unknown.

Based on these findings, we hypothesized that DPSCs are sufficiently responsive to TH for osteogenic differentiation, and the combination of cell-sheet engineering and TH-induced DPSCs would be a simple and convenient method for clinical application to bone regenerative medicine. Therefore, in the present study, we analyzed the effect of TH on the osteogenic differentiation of DPSCs in vitro and the osteogenesis of TH-induced DPSC sheets in vivo.

## Methods

### Cell isolation and culture

This study was performed after receiving written consent from all patients and was approved by the institutional ethics committee of the Faculty of Medicine, Tokyo Medical University, Japan (approval no. 2818). DPSCs were obtained from dental pulp of the wisdom teeth of five healthy patients (18–22 years old) at Tokyo Medical University Hospital. The dental pulp was digested in a solution containing 3 mg/ml collagenase type I (Sigma-Aldrich) for 45 min at 37 °C, and single-cell suspensions were obtained by passing the cells through a 70-μm cell strainer. Human BMSCs were obtained from Takara Shuzo (C12974). The cells were seeded onto 100-mm dishes at a density of 1 × 10^5^ cells and cultured in alpha modification of Eagle’s medium (αMEM; Gibco/BRL) supplemented with 15% fetal bovine serum (FBS; Biowest) and 1% penicillin/streptomycin (P/S; Wako Pure Chemical Industries). All experiments were performed with DPSCs at passage 3 (P3) or P4, and with BMSCs at P4. Cells were cultured in 12-well plates (BD, Franklin Lakes, NJ, USA) and 12-well Upcell (Cell Seed) in osteogenic conditions with or without TH. For osteogenic induction, cells were cultured in αMEM containing 10% FBS, 1% P/S, 10 nM dexamethasone (Dex; Wako Pure Chemical Industries), 10 mM β-glycerophosphate (β-GP; Sigma-Aldrich), and 100 μM l-ascorbate-2-phosphate (AsAp; Wako Pure Chemical Industries) (osteogenic medium (OM)). TH, which was provided by Takeda Chemical Industries (Osaka, Japan), was dissolved in DMSO and added to OM or regular medium (RM) (Dulbecco’s modified Eagle’s medium (DMEM; Gibco/BRL) supplemented with 10% FBS and 1% P/S). For osteogenic induction with BMP, recombinant human bone morphogenic protein 2 (rhBMP-2; Peprotech) was added to the OM (300 ng/ml).

### Flow cytometry analysis

Cells isolated from cultured DPSCs were suspended with 0.25% trypsin, washed with phosphate-buffered saline (PBS), triturated into single cells, and filtered through a 70-μm cell strainer. After suspension, cells were blocked with 10% FBS for 10 min at 37 °C, and incubated with fluorescein isothiocyanate (FITC)-conjugated anti-human CD14, FITC-human CD34, FITC-human CD44, FITC-human CD81, phycoerythrin (PE)-conjugated human CD90, and FITC-human CD105 antibodies (BioLegend) for 90 min at 4 °C. Cells were then washed, and fixed in 4% paraformaldehyde (PFA) for 10 min at 4 °C. Cells that were not treated with fluorescent antibodies were used as controls. The cell surface expression of markers was assessed using a FACS Verse flow cytometer (BD Biosciences). Data were analyzed using the FACS software (FlowJo; FlowJo, LLC).

### Alizarin Red S staining

Cells were rinsed twice with Ca^2+^-free PBS and fixed in 10% formaldehyde in PBS for 10 min at 4 °C. After two washes with distilled water, the cells were stained in 1% Alizarin Red S (Sigma-Aldrich) solution for 15 min at room temperature. The remaining dye was washed out by two washes with distilled water.

### Alkaline phosphatase staining

Alkaline phosphatase (ALP) staining was performed as described previously [15]. Briefly, the cells were rinsed with PBS, fixed in 70% ethanol, and stained for 10 min with 0.01% naphthol AS-MX phosphate (Sigma-Aldrich), using 1% *N*,*N*-dimethyl formamide (Wako Pure Chemical Industries) as the substrate and 0.06% Fast BB salt (Sigma-Aldrich) as a coupler.

### Cell proliferation and viability

Cell proliferation was analyzed using Cell Counting Kit-8 (Dojindo). Cells were seeded at 1 × 10^3^ cells per well in 96-well plates and cultured with or without TH (10^–8^–10^–5^ M). After 72 h the labeling mixture was added to the cells, and cells were incubated for 2 h in a 37 °C/5% CO_2_ incubator. The spectrophotometric absorbance of the samples was measured using a microtiter plate reader at a wavelength of 450 nm.

### Real-time polymerase chain reaction analysis

Total RNA was extracted using Trizol (Invitrogen) and reverse transcription was performed using the QuantiTect Reverse Transcription kit (Qiagen) according to the manufacturer’s instructions. Real-time polymerase chain reaction (RT-PCR) was performed in a Light Cycler 96 (Roche Diagnostics) using THUNDERBIRD SYBR qPCR Mix (Toyobo) under the following conditions: 95 °C for 60 sec and then 45 PCR cycles at 95 °C for 10 sec, 65 °C for 30 sec, and 72 °C for 45 sec. To normalize for differences in the amount of total RNA added to each reaction, *GAPDH* was used as the endogenous control. The primer sequences used are presented in Table 1.

**Table 1:** Sequence information of primers used for quantitative real-time polymerase chain reaction

Gene	Primer sequences (forward and reverse, 5′–3′)
*GAPDH*	GAAGGTGAAGGTCGGAGTCA
	GAAGATGGTGATGGGATTTC
*Runx2*	CAGACCAGCAGCACTCCATA
	CAGCGTCAACACCATCATTC
*Alp*	ATGAAGGAAAAGCCAAGCAG
	ATGGAGACATTCTCTCGTTC
*ColIa1*	GTGCTAAAGGTGCCAATGGT
	CTCCTCGCTTTCCTTCCTCT
*Osteocalcin*	GGCAGCGAGGTAGTGAAGAG
	AGCAGAGCGACACCCTAGAC

### Transplantation of DPSC sheets

Animal experiments were performed according to a protocol approved by the Animal Care and Use committee of the Faculty of Medicine, Tokyo Medical University. Six to eight-week-old male NOD.CB17-Prkdc^scid^/J (NOD SCID) mice (Oriental Yeast) were used. Five mice were used for each experimental group. Each mouse was anesthetized with medetomidine (0.75 mg/kg), midazolam (4.0 mg/kg), and butorphanol tartrate (5.0 mg/ml) via intraperitoneal injection. The skin and the subcutaneous layer were incised, and the calvaria were exposed. Nonhealing and critical-sized (diameter 3.5 mm) calvarial defects were created in the left parietal bone using 3.5-mm disposable biopsy punches (Kai Corporation) [16]. One defect per animal was created. DPSCs were cultured on temperature-responsive dishes in OM with or without TH treatment for 14 days. DPSC sheets were harvested by incubation at room temperature for 30 min, and transplanted into these defects. The skin and subcutaneous layer were then closed by 6-0 nylon sutures. Eight weeks after surgery, calvarias were harvested from euthanized mice.

### Radiological analyses

Micro-CT scanning was performed using a microfocus X-ray CT system (SMX-90CT; Shimadzu) under the following conditions: tube voltage, 90 kV; tube current, 110 μA; and field of view (*XY*), 10 mm. The resolution of one CT slice was 512 × 512 pixels. The three-dimensional construction software package TRI/3D-BON (Ratoc System Engineering) was used for bone morphometric analysis. Bone volume (BV), bone mineral content (BMC), and bone mineral density (BMD) were calculated within a 5 × 5 × 3-mm^3^ cuboid area, in which the circular defect was adjusted to be at the center.

### Histological analysis

For sectioning, mouse calvaria were fixed in 4% PFA/PBS overnight, and decalcified using 10% ethylene-diaminetetraacetic acid (EDTA) solution under gentle shaking for 1 week. Decalcified samples were embedded in paraffin and cut into 5-μm thick sections. Sections were deparaffinized and subjected to hematoxylin and eosin staining and Masson trichrome staining. Sections were observed under a microscope (BZX700; Keyence).

### Statistical analysis

Data were reported as mean ± standard deviation (SD). Statistical significance was evaluated using ANOVA and the Student *t* test for comparison using SPSS 24.0 software (IBM). *p* < 0.05 was considered to indicate a statistically significant difference between two groups.

## Results

### Characterization of DPSCs

**Fig. 1:**
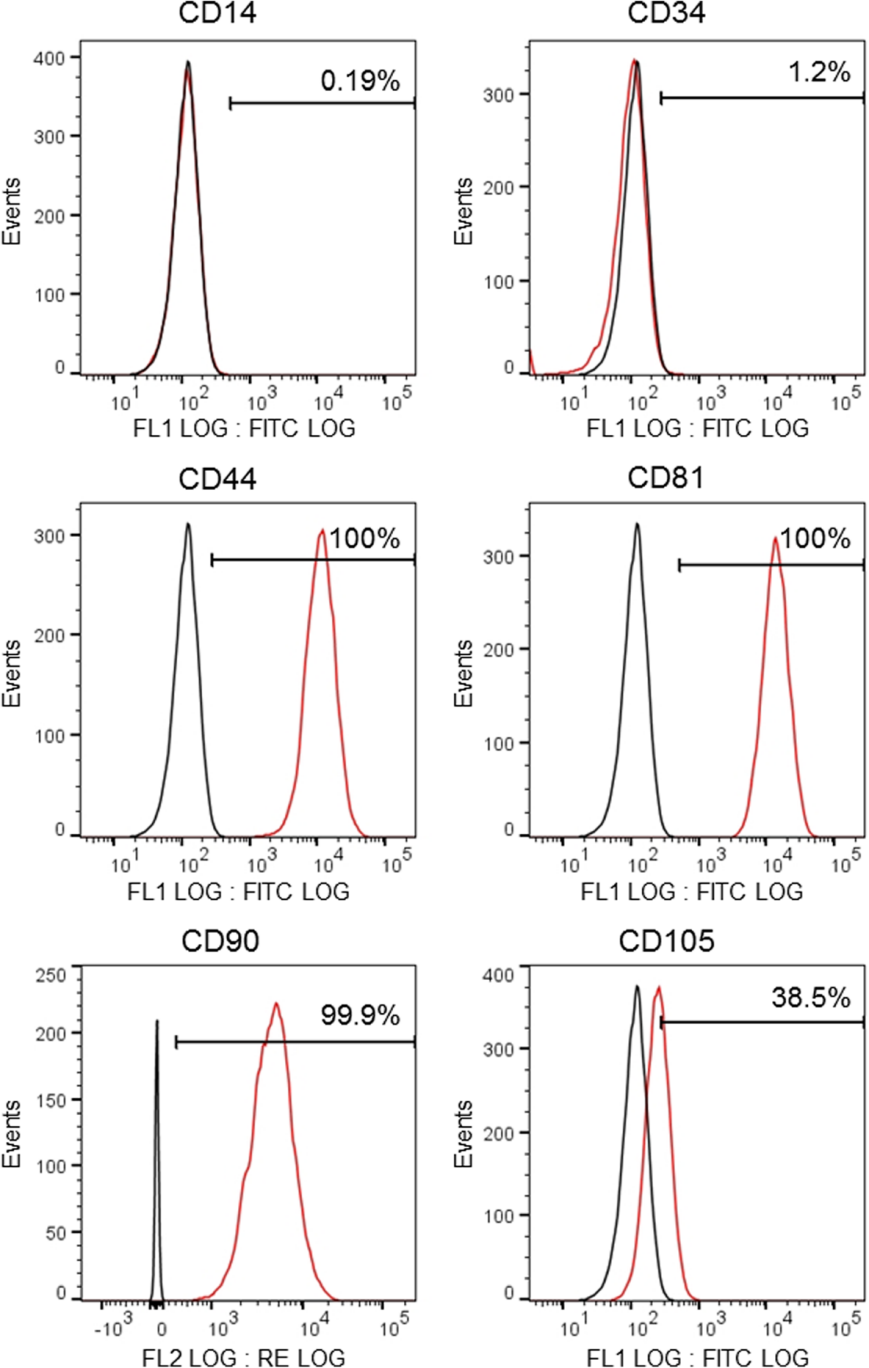
Flow cytometric analysis of DPSCs. DPSCs were cultured at P3 or P4, and surface-stained with FITC-conjugated CD14, CD34, CD44, CD81, and CD105 and PE-conjugated CD90. Fluorescent signals were measured by flow cytometric analysis. Black curve represents control cells, red curve represents cells positive for these markers

To characterize the proportion of DPSCs under our culture conditions, we analyzed the expression of surface markers by flow cytometry. The cells were positive for CD44, CD81, CD90, and CD105, but negative for the hematopoietic stem cell makers CD14 and CD34 (Fig. 1). These data are in accordance with previous studies of DPSC surface expression [3, 17].

### Optimal concentration of TH for the osteogenic differentiation of DPSCs

**Fig. 2:**
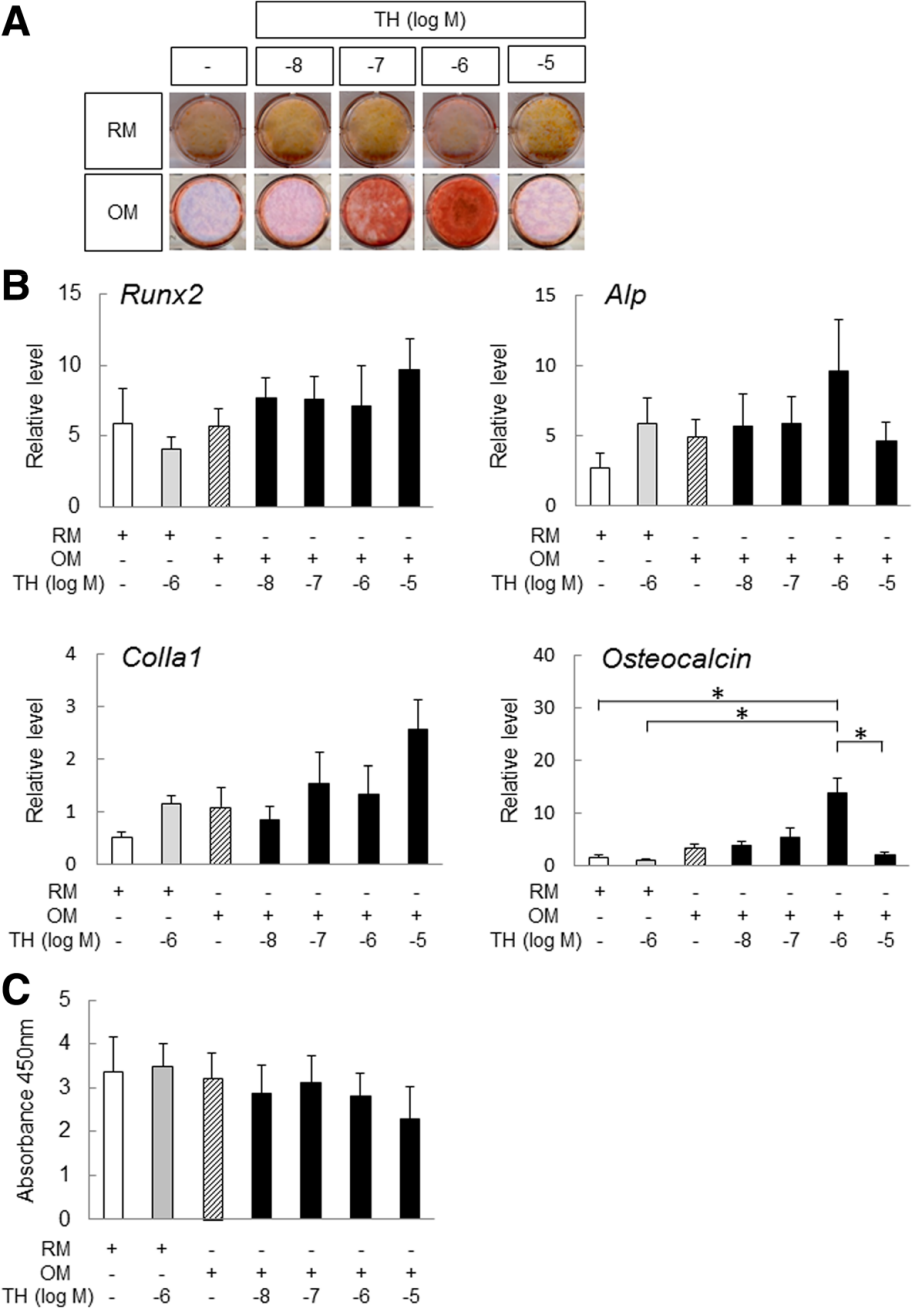
Optimal concentration of TH for inducing osteogenic differentiation of DPSCs. **a** DPSCs cultured in OM or RM with or without TH at concentrations ranging from 10^–8^ to 10^–5^ M. After 21 days, cells were stained with Alizarin Red S to detect matrix mineralization (*n* = 5). **b** After incubation for 72 h, proliferation rates of DPSCs in presence or absence of TH were determined using Cell Counting Kit (*n* = 5). **c** After treatment as in (**a**), RT-PCR performed to measure the expression levels of osteogenic differentiation makers in DPSCs (*n* = 5). Error bars represent SD. Statistical significance determined by one-way ANOVA (*p < 0.05). TH 4-(4-methoxyphenyl)pyrido[40,30:4,5]thieno[2,3-b]pyridine-2-carboxamide, OM osteogenic medium, RM regular medium

To analyze the osteogenic effect of TH in DPSCs, we cultured DPSCs in RM and OM with TH at concentrations ranging from 10^–8^ to 10^–5^ M for 21 days. Osteogenic differentiation was evaluated by matrix mineralization visualized by Alizarin Red staining. Although TH induced no mineralization in DPSCs cultured in RM, TH at 10^–6^ M was found to induce more extensive calcification of DPSCs cultured in OM than at the other concentrations tested (Fig. 2a). TH at 10^–5^ M suppressed matrix mineralization of DPSCs in OM compared with TH at 10^–6^ M. We also analyzed the effect of various concentrations of TH on gene expressions of *Runx2*, an osteoprogenitor maker, *Alp* and *Type I collagen alpha 1* (*ColIa1*), early markers of osteoblast differentiation, and *osteocalcin*, a mature osteoblast marker, by RT-PCR. Although there were no statistically significant differences in the expression levels of *Runx2*, *Alp*, and *ColIa1*, OM with TH at 10^–6^ M significantly upregulated *osteocalcin* expression compared with RM alone and RM with TH at 10^–6^ M (Fig. 2b). To analyze the effect of various concentrations of TH on the proliferation and viability of DPSCs, we used a colorimetric assay for cell proliferation. TH at 10^–5^ M tended to suppress cell proliferation; however, there were no significant differences in proliferation rates between RM and OM with or without TH (Fig. 2c). These data suggest that TH induces the osteogenic differentiation of DPSCs cultured in OM and the optimal concentration of TH for the osteogenesis of DPSCs is approximately 10^–6^ M.

### Osteogenic differentiation of DPSCs by short-term culture with TH

**Fig. 3:**
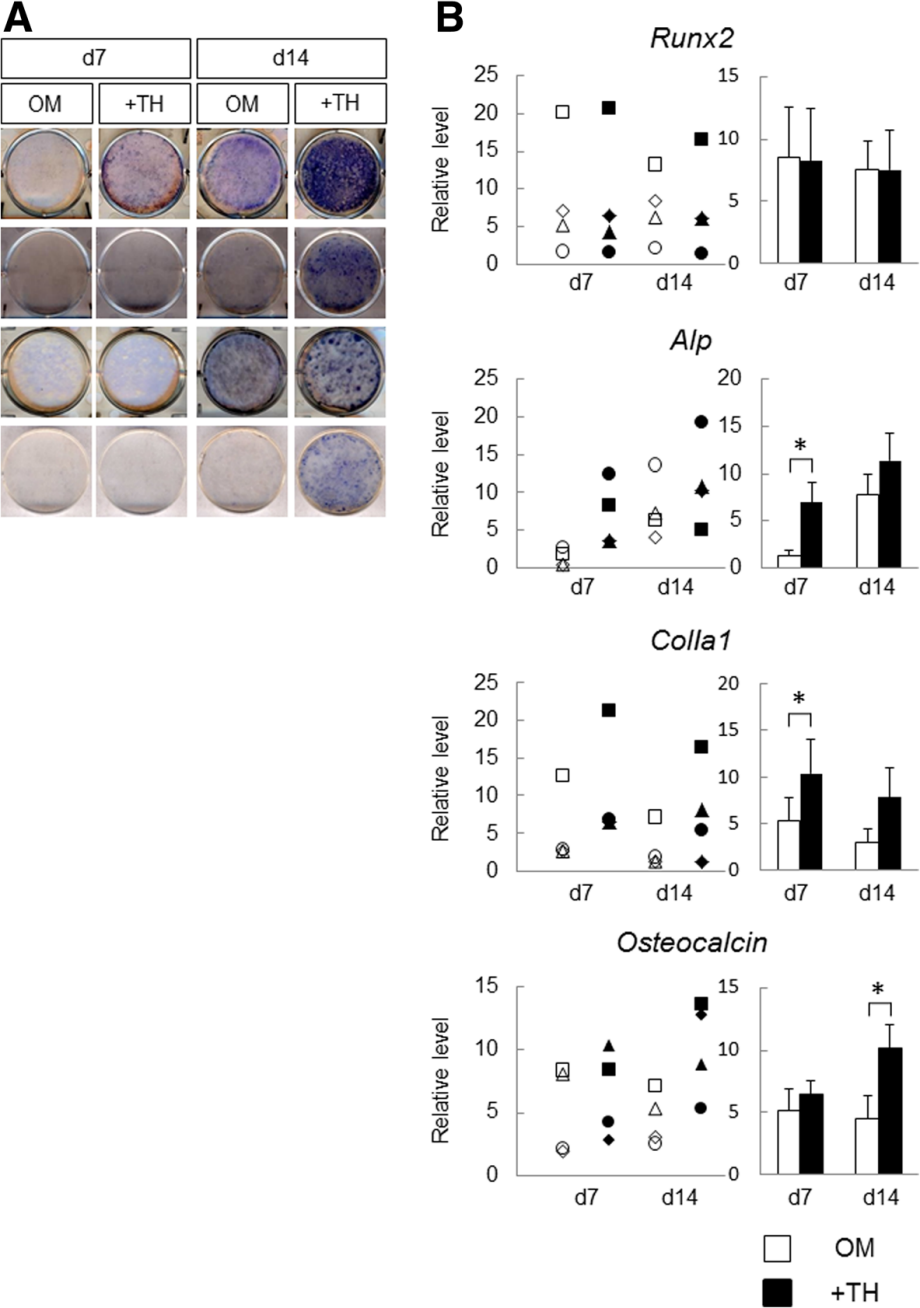
Osteogenic differentiation of DPSCs with TH in short-term culture. **a** DPSCs cultured in OM with or without TH (10^–6^ M). After 7 or 14 days, cells were subjected to ALP staining to detect ALP activity (*n* = 4). **b** RT-PCR performed to measure expression levels of osteogenic differentiation markers in DPSCs cultured with or without TH at day 7 or day 14. Gene expression levels of each sample plotted in the left graph, and the right graph shows the means of four samples. Error bars represent SD. Statistical significance determined by paired Student *t* test (*p < 0.05). d day, OM osteogenic medium, TH 4-(4-methoxyphenyl)pyrido[40,30:4,5]thieno[2,3-b]pyridine-2-carboxamide

We next analyzed the effects of TH for the osteogenic differentiation of DPSCs within a short time. ALP staining showed that TH tended to upregulate and enhance ALP activity of DPSCs at day 7 and day 14 in all samples (Fig. 3a). RT-PCR showed that TH significantly upregulated the expression of *Alp* and *ColIa1*at day 7, and *osteocalcin* was significantly upregulated by TH at day 14. However, TH did not induce *Runx2* mRNA expression at day 7 and day 14. These results are generally consistent with the results of ALP staining and indicate that TH exerts positive effects on the osteogenic differentiation of DPSCs, and its osteogenic effects begin earlier than under conventional osteogenic culture conditions.

### Comparison of the osteogenic differentiation ability of DPSCs and BMSCs in the presence of TH

**Fig. 4:**
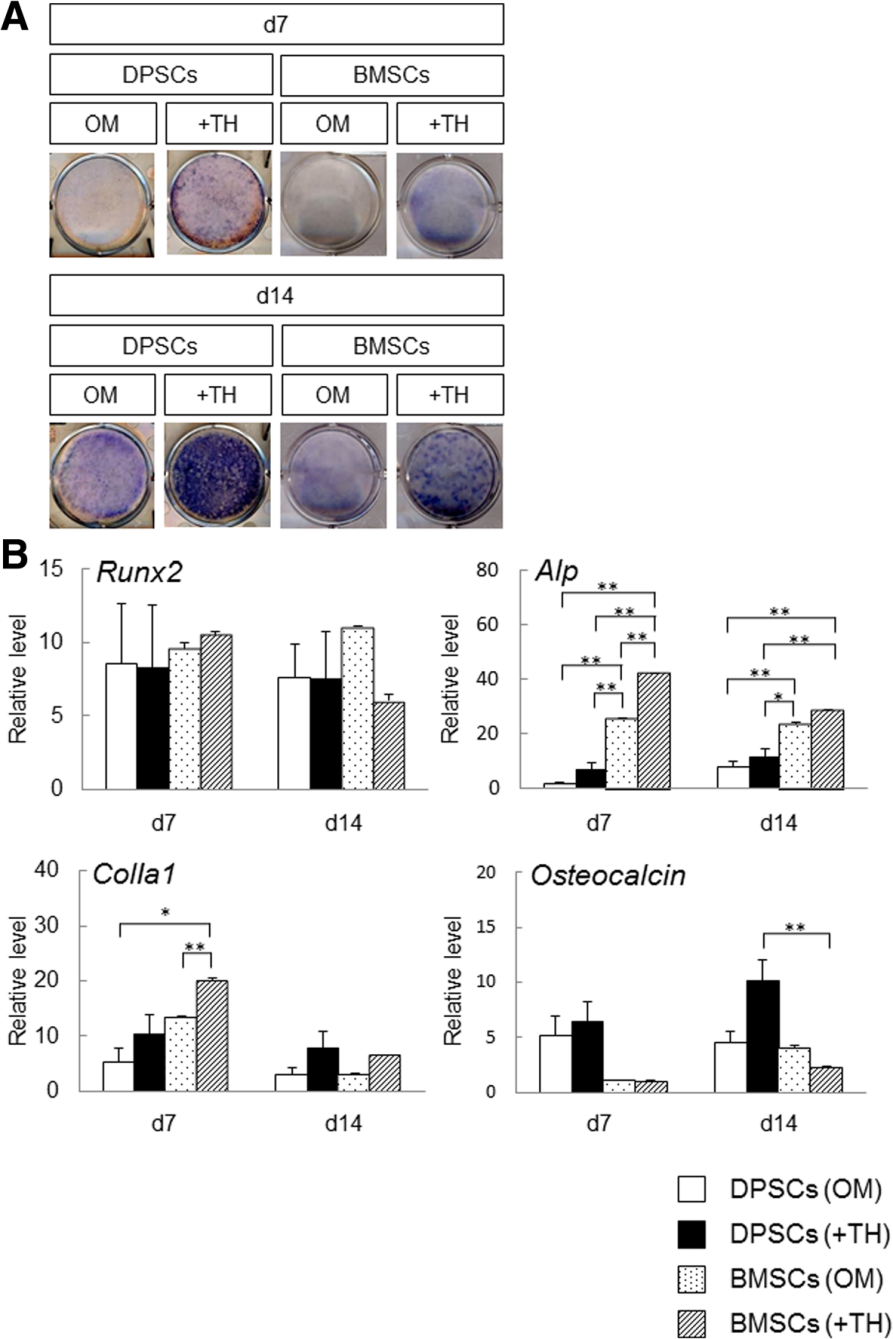
Comparison of osteogenic differentiation ability of DPSCs and BMSCs in the presence of TH. **a** DPSCs and BMSCs cultured in OM with or without TH (10^–6^ M). After 7 or 14 days, cells were subjected to ALP staining to detect ALP activity. **b** RT-PCR performed to measure the expression levels of osteogenic differentiation markers in DPSCs and BMSCs cultured with or without TH at day 7 or day 14. Error bars represent SD. Statistical analyses performed by one-way ANOVA (*p < 0.05, **p < 0.01). Results of DPSCs are from four patients. Results of BMSCs are from three independent experiments. d day, DPSC human dental pulp stem cell, BMSC bone marrow stem cell, OM osteogenic medium, TH 4-(4-methoxyphenyl)pyrido[40,30:4,5]thieno[2,3-b]pyridine-2-carboxamide

To demonstrate the usefulness of TH-induced DPSCs, we compared osteogenic induction abilities between DPSCs and BMSCs in the presence of TH. ALP staining showed that TH enhanced ALP activity in both DPSCs and BMSCs at 14 days (Fig. 4a). RT-PCR analyses showed that TH-induced BMSCs had significantly higher expression levels of *Alp* on day 7 and day 14 compared with TH-induced DPSCs (Fig. 4b). In contrast, TH-induced DPSCs had significantly higher expression levels of *osteocalcin* on day 14 compared with TH-induced BMSCs. There were no statistically significant differences in the expression levels of *Runx2* and *ColIa1* at 14 days between TH-induced DPSCs and TH-induced BMSCs. These results suggest that TH induced osteogenesis in both DPSCs and BMSCs, although the expression pattern in DPSCs induced by TH was different from that in BMSCs within 14 days. Moreover, TH may more strongly promote the maturation of osteoblastic cells from DPSCs than from BMSCs.

### Comparison of the effect of TH and BMP-2 on the osteogenic induction of DPSCs

**Fig. 5:**
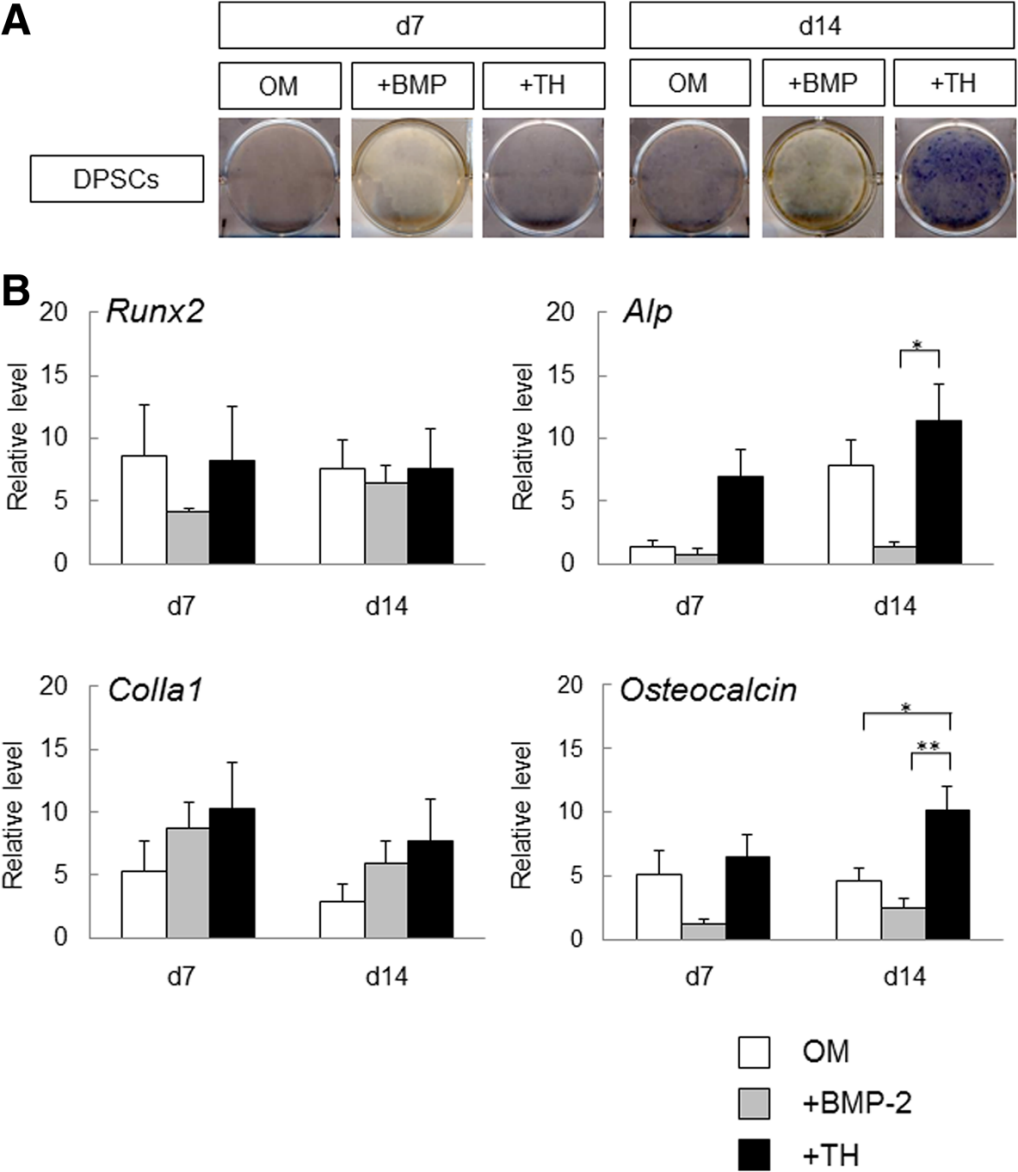
Comparison of effect of TH and BMP-2 on osteogenic induction of DPSCs. **a** DPSCs cultured in OM with BMP-2 (300 ng/ml) or TH (10^–6^ M). After 7 or 14 days, cells were subjected to ALP staining to detect ALP activity (*n* = 4). **b** RT-PCR performed to measure expression levels of osteogenic differentiation markers in DPSCs treated with BMP-2 or TH at day 7 or day 14 (*n* = 4). Error bars represent SD. Statistical analyses performed by one-way ANOVA (*p < 0.05, **p < 0.01). d day, DPSC human dental pulp stem cell, OM osteogenic medium, TH 4-(4-methoxyphenyl)pyrido[40,30:4,5]thieno[2,3-b]pyridine-2-carboxamide, BMP bone morphogenic protein

To determine the effectiveness of TH, we compared the effect of TH and BMP-2 on the osteogenic induction of DPSCs. TH treatment more strongly enhanced the ALP activity of DPSCs compared with OM alone, whereas BMP-2 treatment did not (Fig. 5a). Although there were no statistically significant differences in the expression levels of *Runx2* and *ColIa1* at 14 days, RT-PCR showed that TH significantly upregulated the expression levels of *Alp* and *osteocalcin* at day 14 compared with BMP-2 (Fig. 5b). Furthermore, there was no significant difference in the expression levels of these genes between OM with BMP-2 and OM alone. These results suggest that DPSCs treated with TH more strongly induced osteogenic differentiation compared with BMP-2.

### In-vivo bone regeneration after transplantation of TH-induced DPSC sheets

**Fig. 6:**
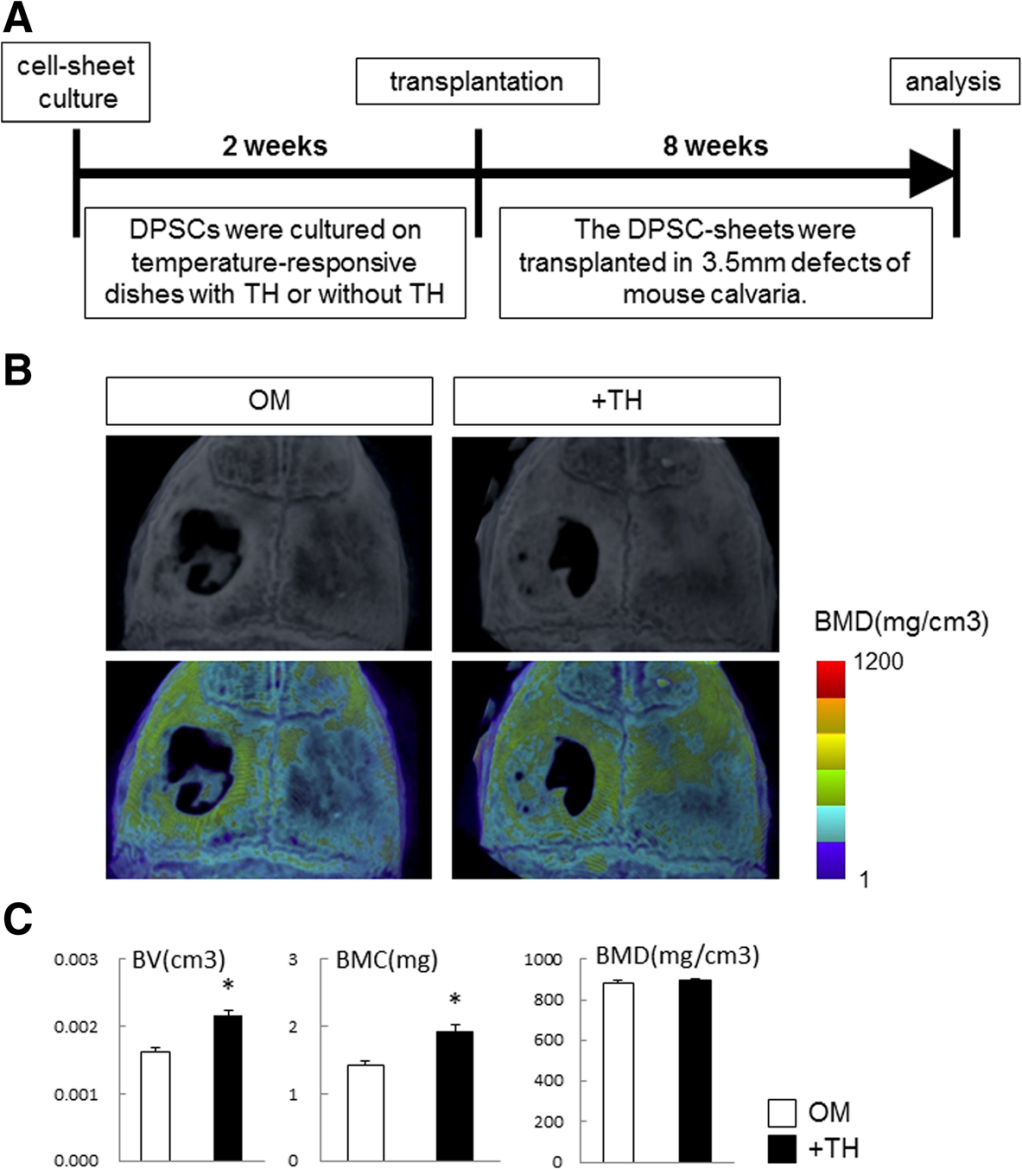
Radiological findings of mouse calvarial bone defects grafted with TH-induced DPSC sheets at 8 weeks after surgery. **a** Experimental procedure. DPSC sheets cultured on temperature-responsive dishes in OM with or without TH (10^–6^ M) for 2 weeks and then transplanted into 3.5-mm defects of mouse calvaria. At 8 weeks after transplantation, calvaria were dissected. **b** Micro-CT images (top) and color-mapped images created from BMD values (bottom). **c** Quantification of BV, BMC, and BMD of 5 × 5 × 3-mm^3^ cuboid samples with a circular defect in the center. Data shown as mean ± SD of five mice per group. *p < 0.05 by Student unpaired two-tailed *t* test. DPSC human dental pulp stem cell, OM osteogenic medium, TH 4-(4-methoxyphenyl)pyrido[40,30:4,5]thieno[2,3-b]pyridine-2-carboxamide, BMD bone mineral density, BV bone volume, BMC bone mineral content

To analyze bone regeneration by TH-induced DPSCs, we cultured DPSC sheets in 12-well temperature-responsive dishes (3.5 cm^2^/well) with TH or without TH (control) for 2 weeks, and then the sheets were folded to fit the 3.5-mm calvarial defects of mice, and were grafted into the defects (Additional file 1: Figure S1). Although minimal bone formation was observed at the edge of the defect, we previously observed that 3.5-mm calvarial bone defects were not covered spontaneously with regenerated bone within 12 weeks of surgery [18]. Therefore, we analyzed bone regeneration in the defects at 8 weeks after transplantation (Fig. 6a). Micro-CT images showed that the DPSC sheets treated with TH induced bone regeneration more extensively than the control sheets (Fig. 6b). Although there were no significant differences in BMD, BV and BMC of the 5 × 5 × 3-mm^3^ cuboid areas containing the circular defects at the center were significantly increased in the TH group compared with the control group (Fig. 6c).

**Fig. 7:**
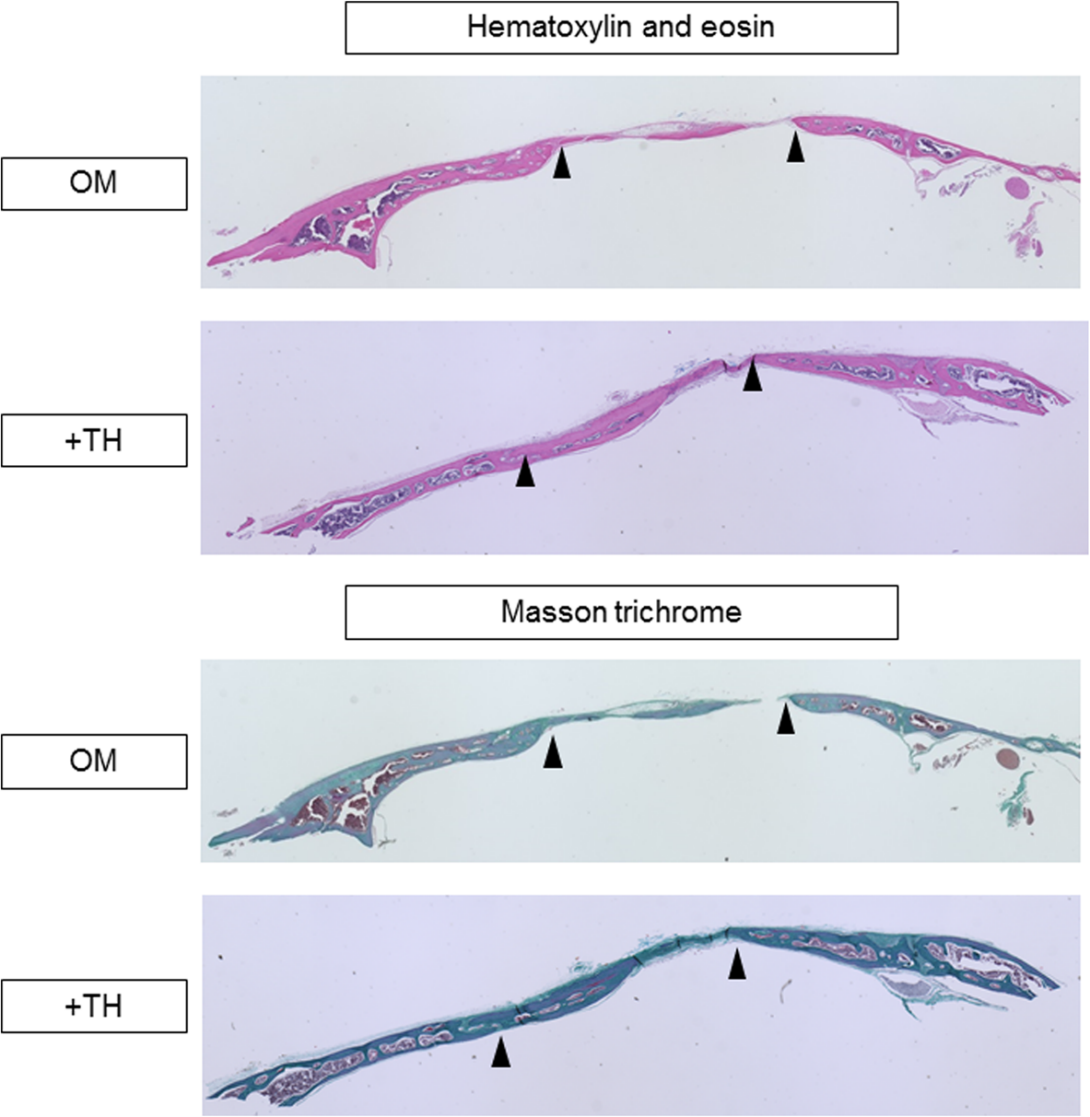
Histological findings of mouse calvarial defects transplanted with DPSC sheets with or without TH treatment. Hematoxylin and eosin staining and Masson trichrome staining of mouse calvarial defects 8 weeks after transplantation of DPSC sheets, treated with or without TH (10^–6^ M). Defect edges indicated by arrowheads. OM osteogenic medium, TH 4-(4-methoxyphenyl)pyrido[40,30:4,5]thieno[2,3-b]pyridine-2-carboxamide

To investigate this further, we analyzed new bone formation by histological analyses. Hematoxylin and eosin staining showed that new bone formation was apparent in the defects treated with TH-induced DPSC-sheet compared with control defects (Fig. 7). Masson trichrome staining demonstrated that the defect area was occupied by osteoid matrix in the TH group. Thus, DPSC sheets treated with TH to induce osteogenic differentiation formed new bone tissue without requiring scaffold or growth factors.

## Discussion

In the present study, we analyzed the osteogenic effects of TH on DPSCs and the in-vivo osteogenesis ability of TH-induced DPSCs using cell-sheet technology. We first demonstrated that the optimal concentration of TH on the osteogenic differentiation of DPSCs is approximately 10^-6^ M. Next, we found that TH induces the osteogenic differentiation of DPSCs after a short culture time. Furthermore, we demonstrated that TH induces the osteogenesis of DPSCs more strongly than BMP-2, and induces the osteogenesis of both DPSCs and BMSCs. Finally, DPSC sheets treated with TH enabled successful bone healing in a mouse calvarial defect model.

DPSCs demonstrate higher clonogenic and proliferative potential than BMSCs [2], and can easily be obtained from tooth extraction surgery. DPSCs are expected to become an important cell source for regenerative medicine and tissue engineering. When using DPSCs for regenerative medicine, it is important to obtain a sufficient number of cells maintaining multipotency. However, the number of cells obtained from dental pulp is limited. Physiological secondary dentin and pathological tertiary dentin are formed by odontoblasts with age, resulting in a decrease in pulp tissue volumes [19]. In addition, MSCs progressively lose their multipotency and proliferative potential with age or during long-term culture [20]. To overcome these problems and to apply DPSCs in the clinical setting, culture methods that enable the proliferation and differentiation of DPSCs at high efficiency are required. In the present study, to develop a novel approach for bone regenerative medicine, we analyzed the osteogenic differentiation of DPSCs using TH, which is a small osteogenic small molecule.

We reported previously that TH induces osteogenic differentiation of preosteoblastic cells and mesenchymal cells in vitro and in vivo [13, 14, 21], and that the optimal concentration for producing the osteogenic effects of TH on MC3T3-E1 and C3H10T1/2 cell lines is 10^–6^ M [14]. In DPSCs, Alizarin Red staining showed that TH at 10^–6^ M induced more extensive calcification than the other concentrations tested, and RT-PCR showed that TH at 10^–6^ M significantly upregulated *osteocalcin* expression compared with the other conditions. Although OM with TH at 10^–5^ M tended to suppress cell proliferation, there were no significant differences in proliferation rates between RM and OM with or without TH. We reported previously that TH at 10^–5^ M had a lower osteogenic effect on MC3T3-E1 cells than TH at 10^–6^ M, probably due to the toxicity of TH [14]. When DPSCs were cultured in RM, TH failed to induce osteogenic induction. We also reported that the osteogenic effect of TH in mouse embryonic stem cells required Dex, β-GP, and AsAp [14]. These data suggest that the optimal concentration of DPSCs for osteogenesis is approximately 10^–6^ M, and that OM is essential for the osteogenic effects of TH in DPSCs.

Compared to conventional OM, TH significantly upregulated the expression levels of *Alp* and *ColIa1* in DPSCs on day 7 of culture. Moreover, the late osteoblast marker *osteocalcin* was significantly upregulated by TH on day 14. ALP staining showed that TH treatment enabled ALP activity of DPSCs to be detected much earlier than the control in all experimental samples. For clinical application, the culture period of transplant cells should be minimized to prevent contamination, such as with viruses and bacteria, as well as to reduce the cost of culturing, and to use less auto serum. In this regard, we expect that TH will shorten the culture period for the osteogenic differentiation of DPSCs.

The properties of cell sheets from various cell sources have been analyzed previously regarding bone regeneration [22, 23]. Because cell sheets are flexible and simple to transplantation, cell-sheet technology may replace conventional bone graft methods in the future. Our DPSC sheet transplantation method combined with TH achieved bone regeneration without the requirement for scaffolds or growth factors. Previous studies have shown that DPSCs have a higher ability to prevent T-cell alloreactivity and immunosuppressive activity compared with bone marrow-derived MSCs [24], and DPSC allografts enabled successful bone regeneration without requiring any immunosuppression in experimental animals [25]. Therefore, the induction of osteogenic differentiation in autologous or allogeneic DPSCs by TH may help establish a more efficient bone regeneration method, and our convenient method of DPSC sheets in combination with TH may overcome some of the problems associated with current bone regeneration methods, such as the invasion or stress of harvesting autologous bone and MSCs, ethical and safety issues concerned with allogeneic bone or recombinant proteins, and infection of artificial materials.

One limitation of this study is that the mechanism underlying osteogenic induction by TH in DPSCs remains unclear. We reported previously that TH induced the osteogenic differentiation of preosteoblasts in a BMP-dependent manner [4]. However, a combination of TH and BMP-2, but not TH alone, induced osteogenic differentiation in mouse embryonic stem cells and in mouse and human dermal fibroblasts. In contrast to the osteogenic effect of BMP-2 on BMSCs [8], in the present study BMP-2 did not induce osteogenesis in DPSCs. Furthermore, TH alone induced osteogenesis in both DPSCs and BMSCs, although the gene expression pattern induced by TH in DPSCs was different from that in BMSCs. The differences of the effects of TH and BMP-2 between DPSCs and BMSCs may be owing to the different origins of the cells. BMSCs are derived from the mesoderm, whereas DPSCs originate from cranial neural crest cells and express markers characteristic for cranial neural crest-derived stem cells, such as nestin, HNK-1, P75, and glial fibrillary acidic protein [26]. With regard to the osteogenic mechanisms of TH, we are planning to investigate the direct molecular target of TH using a helioxanthin derivative which has a functional moiety interacting with magnetic beads through amide bonding, and hence can be purified and also retains osteogenic ability at a comparable level to that of TH. For clinical application of DPSCs using TH, we should analyze not only the molecular mechanisms involved in the effect of TH on DPSCs but also its bioactivity and toxicity.

## Conclusions

We demonstrated that TH induces the osteogenic differentiation of DPSCs in short-term culture, leading to shortened culture duration for osteogenic differentiation of DPSCs. Furthermore, TH-induced DPSC sheets promote osteogenesis in vivo. Thus, this transplantation method of DPSCs with TH treatment may be useful for bone regenerative medicine in terms of safety and convenience.

## Additional file

Additional file 1: Figure S1. Mouse calvarial defect model and procedure for implantation of DPSC sheets into a bone defect. (**A**) Gross appearance and micro-CT image of mouse calvarial defect model before transplantation. (**B**) Procedure for implanting DPSC sheets into the mouse calvarial bone defects (TIF 318 kb)

## Abbreviations

ALP: Alkaline phosphatase
AsAp: l-Ascorbate-2-phosphate
BMC: Bone mineral content
BMD: Bone mineral density
BMP: Bone morphogenic protein
BMSC: Bone marrow stem cell
BV: Bone volume
*ColIa1*: *Type I collagen alpha 1*
Dex: Dexamethasone
DPSC: Dental pulp stem cell
EDTA: Ethylene-diaminetetraacetic acid
FITC: Fluorescein isothiocyanate
β-GP: Beta-glycerophosphate
αMEM: Alpha modification of Eagle’s medium
MSC: Mesenchymal stem cell
NOD SCID: NOD.CB17-Prkdc^scid^/J
OM: Osteogenic medium
PBS: Phosphate-buffered saline
PE: Phycoerythrin
PFA: Paraformaldehyde
P/S: Penicillin/streptomycin
rhBMP-2: Recombinant human bone morphogenic protein 2
RM: Regular medium
RT-PCR: Real-time polymerase chain reaction
SD: standard deviation
TH: 4-(4-Methoxyphenyl)pyrido[40,30:4,5]thieno[2,3-b]pyridine-2-carboxamide
