# Supplementary figures and images for: Bone regeneration by human dental pulp stem cells using a helioxanthin derivative and cell-sheet technology
Source: Stem Cell Res Ther. 2018 Feb 1;9:24. doi: 10.1186/s13287-018-0783-7 (PMC5796442; doi:10.1186/s13287-018-0783-7)

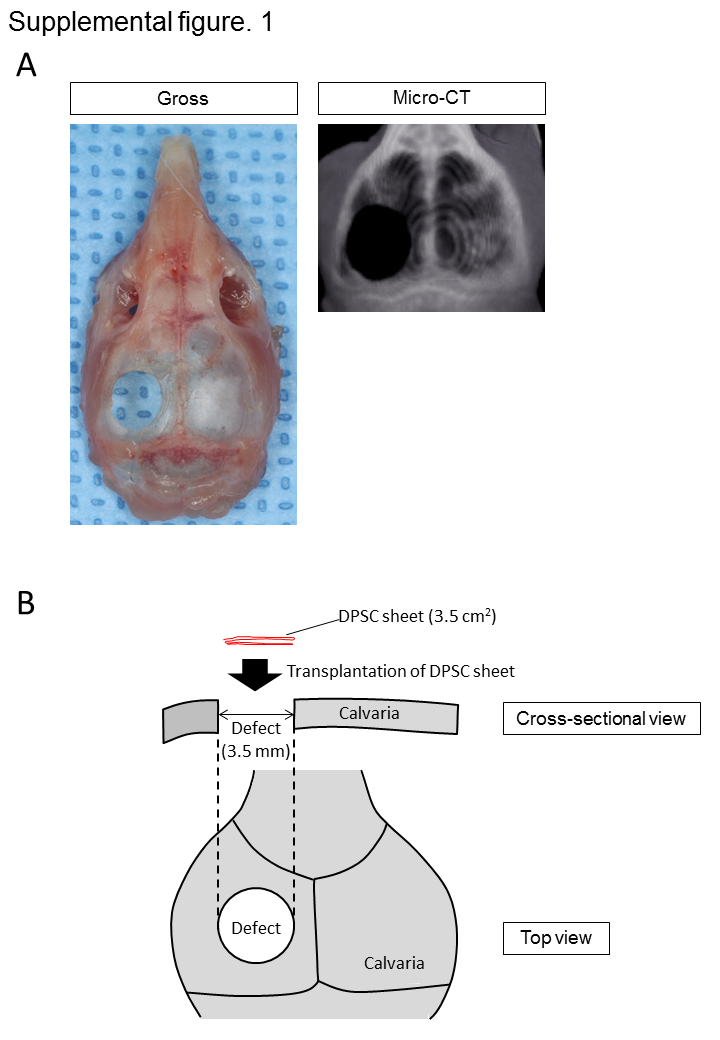

Supplement: Additional file 1: Figure S1. — Mouse calvarial defect model and procedure for implantation of DPSC sheets into a bone defect. (A) Gross appearance and micro-CT image of mouse calvarial defect model before transplantation. (B) Procedure for implanting DPSC sheets into the mouse calvarial bone defects (TIF 318 kb) [file 13287_2018_783_MOESM1_ESM.tif]
